# To G. M. Burrows, Esq. Chairman of the Committee of Apothecaries, &c. &c. &c.

**Published:** 1813-06

**Authors:** 


*For the Medical and Physical Journal.*

*To* G. M. Burrows, *Esq. Chairman of the Committee of Apothecaries, &c. &c. &c.*

Sir,

A LETTER having lately appeared, addressed to Sir Francis Milman, bearing the signature of "One of your Committee," and taking it for granted, from the evident ingenuity and zeal of its author, that all the strong points and leading arguments of that writer, and of the advocates of his cause, are there concentrated, I take the liberty through you, Sir, as the organ of that committee, to offer some animadversions upon it.

It is much to be regretted that the bill lately submitted to the House of Commons, for the improvement, &c. of the condition of the Apothecaries, &c. did not arrive at a stage where its merits might be more amply discussed; for, although fraught with absurdity, and containing in almost every paragraph matter of the most objectionable nature, one important advantage must have accrued, that the station, rank, and *duties*, of the different departments of the profession, would have been accurately defined, and the minds of medical men, as well as of the public, have been set at rest upon that most interesting subject. It strikes me, Sir, that the ground of the present controversy might be much narrowed, or possibly its cause entirely removed, by a plain answer to a plain question—"Have the Apothecaries any legal authority *to prescribe* medicine for the sick?"—I am aware that the writer above alluded to has in several passages of his letter assumed and reasoned upon the affirmative; but assertion must not go for proof, and I call upon that gentleman, and all the supporters of his opinions, to produce any act of the legislature, or any document from the College of Physicians, expressive of, or implying in any shape, a sanction or recognition of that authority: on the contrary, we are indebted to the industry and research of this writer, for furnishing us with numerous instances where prosecutions were commenced, and fines levied on apothecaries, and other unqualified persons, for "daring to compound and dispense medicines without the prescription of the physician."* And he proceeds to state, that after the reign of Charles the First, "the interdictions against apothecaries and surgeons to practise medicine gradually became obsolete."

These facts serve at least to prove that such prohibitions

* See page 9.

and and legal incapacities did exist: it therefore becomes necessary for his purpose, to show at what time, or upon what occasion, they were nullified and repealed. What he seems most to rely upon is an act of the sixth of William the Third, which was made perpetual in the ninth of George the First, to exempt apothecaries from certain parish and ward offices, because, being so engaged, "they could not perform the trusts reposed in them as they ought, nor *attend* the sick with such diligence as is required." The construction which this writer has put on these words, exhibits a melancholy proof how far a good understanding may be warped by an overweening attachment to a particular object. Had he considered the passage with that good sense which I believe is natural to him, "Si mens non læva fuisset," I am persuaded he would have found that a conclusion diametrically the reverse of his own would have been more consonant to just and legitimate induction.—"Nor attend the sick with such diligence"—Sir, I am far from meaning an indecent comparison, but I will say, that it is also the duty of a nurse to attend the sick, yet no man will be guilty of such an outrage on propriety as to assert that she has a right to prescribe for them.

Again, he says, (p. 10) that the right of the apothecary to practise (that is, to prescribe) has even been admitted by what may be termed authority, for that the outlines of a plan circulated some years ago by the College of Physicians, provided for "the proper medical education of the apothecary before he can be allowed to practise." See Appendix B. Here also we observe the same want of candour: why did he not add the remainder of the paragraph, and render the sense complete, "as an apothecary of the United Kingdom?" I have some reason to be acquainted with the sentiments of that learned and respectable body, and I take upon me to affirm, that they never entertained an idea of giving countenance to the arrogant and unreasonable pretensions set up by the apothecaries, &c. For the due understanding of that plan, I would refer to the document itself, being perfectly convinced that the most superficial glance will satisfy any unprejudiced person of what I have stated. In clause 8, it says, "that apothecaries by their education *entitled to act as such,* shall be examined by their respective corporation or company as to their qualification so to do;" and, in the clause immediately preceding, (Appendix, clause 4) the outline declares "that no chemist, druggist, or vendor of medicine, shall be permitted to compound or dispense medicines in a retail manner, unless he should have had the regular education of an apothecary, or be otherwise legally authorised authorised so to do." Will it then be maintained by any rational being, that the College, to whom are delegated the powers and authorities for "overseeing, ruling, and governing, the faculty of physic, for the benefit and protection of the community," ever had it in contemplation to give a sanction to the claims now advanced, which so vitally affect the safety and well-being of that community, as well as the honor, credit, and dearest interests, of their own members? that they would recommend (as this writer would have us believe) that apothecaries should undergo a previous examination touching the knowledge of diseases and capability of prescribing, by "their respective corporation or company," a description of persons whom he has characterised, "though highly respectable in themselves, as merely a *trading company?*" Credat Judæus.—Really, sir, such a supposition is truly absurd, and I have to offer you many apologies for wasting so much of your time in its refutation.

The instance which the "Member of the Committee" has adduced of King Henry the Eighth having settled a pension of forty marks on John Soda his apothecary for his attendance on the Princess afterwards Queen Mary, is not more relevant: still it was for his *attendance*, and it would appear that that royal person herself entertained an opinion of John's services very different from that of her father, for we find that in the third year of her reign, "surgeons and apothecaries were prohibited the practising of physic." The page of history has branded the name of that princess with many of the worst vices: it remained for the "Member of the Committee" to stigmatise her with *one* which some moralists have considered as a consummation of them all.*

With much of what this gentleman has said in respect to the state of physic on the continent, I entirely agree, throughout its whole extent, "A Gadibus usque Auroram et Gangen." P. 38, "The physician is *the only prescriber* in medical cases." P. 40, "The continental apothecary is a mere compounder, resembling in a great degree the compounding chemist and druggist of this metropolis;" and he might have added, would be liable to the severest penalties if he presumed to trench upon that department of medical practice which the whole letter and spirit of their laws suppose him incompetent to. I do not hesitate to declare my opinion, that that mutual cordiality and reciprocal attachment which the general interests of the profession imperatively require, will never be restored until arrangements in many of their leading characters similar to those on the continent

* Ingratum si dixeris omnia dixeris.

be be established; but, when I say this, I express rather my wishes than my hopes: I know the aspiring and inadmissible pretensions of some, certainly not the most numerous nor intelligent of the apothecaries; I know that so far are they from being content with that "important situation and responsibility" they have already acquired, which, though never formally and regularly granted, has been tacitly and indulgently permitted, that nothing will satisfy their inordinate and preposterous ambition but the extinction of the higher departments of the profession, and the elevation of themselves in their stead. Can any man who has the least regard for order and subordination, listen without a mixture of contempt and indignation to the tone they have recently assumed? "The sub or minor physician," "the physician in ordinary," and other such new-fangled ridiculous nonsense. "Optat ephippia bos piger." That such are the views of these "amphibological" gentlemen appears to me as clear as the sun at noon, from what I have been obliged to hear from their own lips, as well as from several passages in the pamphlet under my immediate consideration. They well know that the first and most effectual step towards the annihilation of any description of men, is to disparage and depreciate them in the opinion of the public, and conformably to this principle, towards the conclusion, besides a general attack on the college in their collective capacity, we find, in various parts of the work, the following gross and unfounded insinuations against individuals:—P. 20, "And it is not surprising that such men (physicians) should feel alarmed at meeting an apothecary in consultation, by whom their defects would be readily perceived." P. 27, "Many of those (apothecaries) have received *educations* not inferior to *those* of the majority of physicians." Again, p. 32, "It is barely possible also that some individuals of the college may feel jealous of the superior qualifications which the establishment of such a superintending court is likely to confer on the apothecaries."

This letter has already exceeded the limits I originally intended, but I find the subject grow upon me, and that many important points still remain untouched. The consideration of these I am obliged, from unavoidable circumstances, to defer for the present, but shall certainly take an early opportunity of reverting to them, either in a *second letter to Sir Francis Milman, or as a continuation of the present one to yourself. I have the honor of being, Sir,

Your's, &c.

*April* 15, 1813. CENSORINUS.

* See Medical Journal for February.

